# Synthesis and Antibacterial Properties of Novel ZnMn_2_O_4_–Chitosan Nanocomposites

**DOI:** 10.3390/nano9111589

**Published:** 2019-11-09

**Authors:** Rajiv Gandhi Packirisamy, Chandramohan Govindasamy, Anandhavelu Sanmugam, K. Karuppasamy, Hyun-Seok Kim, Dhanasekaran Vikraman

**Affiliations:** 1 Research and Development Centre, Bharathiar University, Coimbatore 641046, India; p.rajivgandhi@gmail.com (R.G.P.); chandramohan1956@gmail.com (C.G.); 2 Department of Chemistry, Vel Tech Multi Tech., Chennai 600062, India; sranand2204@gmail.com; 3 Division of Electronics and Electrical Engineering, Dongguk University-Seoul, Seoul 04620, Korea; karuppasamyiitb@gmail.com (K.K.); hyunseokk@dongguk.edu (H.-S.K.)

**Keywords:** ZnMn_2_O_4_, Mn_3_O_4_, chitosan, nanocomposites, antibacterial

## Abstract

The development of productive antibacterial agents from nontoxic materials via a simple methodology has been an immense research contribution in the medicinal chemistry field. Herein, a sol–gel one-pot reaction was used to synthesize hybrid composites of hausmannite–chitosan (Mn_3_O_4_–CS) and its innovative derivative zinc manganese oxide–chitosan (ZnMn_2_O_4_–CS). Fixed amounts of CS with different metal matrix *w*/*v* ratios of 0.5%, 1.0%, 1.5%, and 2.0% for Mn and Zn precursors were used to synthesize ZnMn_2_O_4_–CS hybrid composites. X-ray diffraction analysis indicated the formation of polycrystalline tetragonal-structured ZnMn_2_O_4_ with a CS matrix in the hybrids. Fourier-transform infrared spectroscopic analysis confirmed the formation of ZnMn_2_O_4_–CS hybrids. Detailed investigations of the surface modifications were conducted using scanning electron microscopy; micrographs at different magnifications revealed that the composites’ surface changed depending on the ratio of the source materials used to synthesize the ZnMn_2_O_4_–CS hybrids. The antibacterial activity of the Mn_3_O_4_–CS and ZnMn_2_O_4_–CS composites was tested against various bacterial species, including *Bacillus subtilis*, *Escherichia coli*, *Salmonella typhi*, and *Pseudomonas aeruginosa.* The zone of inhibition and minimum inhibitory concentration values were deduced to demonstrate the efficacy of the ZnMn_2_O_4_–CS nanocomposites as antibacterial agents.

## 1. Introduction

Since antiquity, even after many revolutionary clinical discoveries, human societies have faced tremendous obstacles posed by infection, which costs billions of dollars per year because of clinical difficulties and the lengthening of patient recovery times [1,2]. In cases of infection, medical strategies with respect to transplants frequently necessitate a secondary surgical treatment or procedure, which increases medical expenses for patients [3]. The advent of effective and nontoxic antibiotics-undoubtedly one of the supreme achievements in the history of medicine—has brought immense benefits [4]. The ability to cure infections in the preliminary stage without identifying the root cause of the relevant pathogen has preserved countless lives and enabled numerous advances in modern clinical medicine, such as organ transplantation, surgery, chemotherapy, and premature-infant care [5].

The term *antibiotics* was first used by Selman Waksman in 1941 to describe the antimicrobial agents created by certain microorganisms [6,7]. Antibacterial agents are required to kill microbes or slow their development without themselves being toxic to the adjacent tissues. Antibacterial agents, a subcategory of antibiotics, can be produced naturally by fungal sources (e.g., gentamicin, cefamycins, and benzylpenicillin) and synthetically by the derivatization of natural products. In general, natural antibacterial agents tend to be more harmful than synthetic ones. In addition, synthetic antibacterial agents can be designed to have better efficiency and lower toxicity; hence, they have advantages over natural antibiotics [8].

The rise of pathogenic and putrefaction bacteria resistant to antimicrobial agents has become a major health concern. Biomedical researchers are therefore exploring pathways to improve existing antimicrobial agents. Antibacterial agents are bactericidal; they kill microorganisms by penetrating the wall or membrane of a cell of the pathogen and rendering it bacteriostatic (i.e., inactive) or by preventing its growth. The inhibition phenomenon of bacteriostatic agents involves preventing protein production or impeding bacterial vitality pathways [9,10]. Bacteriostatic agents obstruct pathogenic bacteria growth; however, the distinction between bacteriostatic and bactericidal property is not always clear [11].

Antibacterial agents are used in various applications such as water disinfection, medicine, the textile industry, and food packaging [12,13]. The recent nearly unrestricted use of antibiotics has intensified the development of antibiotic-impervious genes in several bacteria. Well-renowned antimicrobial agents have revealed the resistance of one microorganism species or another [14]. Bacterial microorganisms are incessantly transformed, triggering antibiotic resistance. This behavior is the result of various mechanisms; however, the primary factor is bacterial-produced enzymes with the ability to alter, damage, or deactivate antibiotics [10,15]. The delayed penetration leads to antibiotic disengagement before the antimicrobial agent can perform its function, which can lead to infections that extend to other organs; preventing such infections is expensive because of the requirement of maintaining a sterile environment, although failure to do so can lead to an increase in mortality [16]. Hence, the development of antibacterial agents that are inexpensive, nontoxic, and robust is critical to addressing the problem of antibiotic-resistant pathogens and is an imminent concern [17].

Nanomedicines such as nanomaterials provide durable, targeted, and comprehensive antimicrobial activity at lower doses than conventional antibiotics [16]. The nanomaterials enable durable antimicrobial interactions with pathogens because of their small dimensions compared with those of bacteria and their low volume-to-surface-area ratio. Nanomedicines can destroy or inhibit the growth of genes by infiltrating the cell walls of bacteria, causing the condensation of DNA molecules and preventing reproduction or growth [18]. The antimicrobial activities of nanomaterials vary dramatically depending on the morphological and physicochemical characteristics of the nanomaterials [19,20]. In addition, nanomaterials consisting of metal ions can be directly linked to the thiol groups of the cysteine amino acid rather than to the sulfur of proteins, inducing a huge alteration of the active sites and leading to cell mortality [4,21]. The antibacterial activity of nanomaterials can originate from the harmful metal ions produced from the termination of metals and oxidative stress through creation of reactive oxygen species (ROS) on the nanomaterials’ surface [21,22]. The positive surface charge of nanomaterials enables their binding to bacteria with a negative surface charge, which enhances their bactericidal properties [23,24]. In addition, the size and shape of nanomaterials strongly affects their antimicrobial activity [25,26].

Chitin, a naturally abundant biopolymer, can be obtained from crustacean shells and fishing-industry wastes [27]. Chitosan (CS) is a deacetylated form of chitin and a decomposable polysaccharide that contains β-linked d-glucosamine polymer [2]. Numerous authors have claimed that CS exhibits inherent antibacterial behavior against various yeasts, bacteria, and fungi; this behavior is attributed to the positive charge of its amino groups, which electrostatically binds the CS to the surface of a cell membrane to deactivate the cell’s enzymes [27,28,29].

The particle size and material type used to prepare nanoparticles are two important factors in tuning their antibacterial effectiveness [19,21]. However, such nanoparticles cause apprehension about their toxicity, which is related to their heavy metal content and their accretion with the human body [30,31]. Nanostructures of various metal oxides such nickel(II) oxide, iron(II) oxide, silver oxide, titanium(IV) oxide, copper oxide, tin oxide, zinc oxide (ZnO), and manganese(II,III) oxide (Mn_3_O_4_) have been intensively investigated as potential antibacterial systems [32,33]. Indeed, ZnO nanostructures have been reported to show antibacterial activity toward various harmful organisms that are tolerant of high temperatures and high pressures [22]. The greater antibacterial activity of ZnO nanostructures compared with that of ZnO microstructures is attributable to the enhanced surface area of the nanostructures [34]. Mn_3_O_4_ is one of the most stable oxides and is used effectively as a catalytic material for the oxidative destruction of volatile organic compounds [35]. In addition, medicinal science has revealed that nanocrystalline Mn_3_O_4_ is an excellent antioxidant and antibacterial system [35,36,37]. Various metal oxide hybrid systems have been intensively studied, including by our research group, as promising materials for antibacterial applications because of their synergistic behaviors [4,29,38,39].

In the present work, we focus on the sol–gel synthesis of hybrid systems of zinc manganese oxide–CS composites (ZnMn_2_O_4_–CS) with various concentrations of metal oxides. A comprehensive examination of antibacterial properties was carried out by zone-of-inhibition and minimum-inhibitory-concentration (MIC) studies against *Bacillus subtilis* (*B. subtilis*), *Escherichia coli* (*E. coli*), *Pseudomonas aeruginosa* (*P. aeruginosa*), and *Salmonella typhi* (*S. typhi*).

## 2. Materials and Methods

### 2.1. Chitin Refinement

The CS was synthesized from chitin through the deacetylation method [4,40]. Initially, crab shells were collected, cleansed in seawater, and then dehydrated in sunshine for 1 day. The air-dried shells were ground, and then 50 g of the shell powder was dissolved in hydrochloric acid (5%) solution with continual stirring at room temperature for 2 h. The resultant was subsequently cleansed using distilled water to eliminate calcium chloride and acid impurities through demineralization. The demineralized powders were stirred in sodium hydroxide (NaOH) (5%) solution for 24 h for de-proteinization. The acquired deposit was cleaned with de-ionized liquid till the wash solution was pH-neutral. The resultant chitin powder sample was dehydrated in an oven at 60 °C for 1 h.

### 2.2. Mn_3_O_4_–CS Synthesis

Manganese chloride (MnCl_2_) with 0.5% (*w*/*v*) and purified chitin (0.25 g) liquefied 2% acetic acid (AA) were mixed in a beaker with constant magnetic stirring; the beaker solution was then transferred to hotplate at 70 °C for 2 h. Freshly prepared NaOH solution (45% *w*/*v*) was mixed dropwise till a brown precipitate formed, and the precipitate was allowed to settle as a residue at room temperature for 24 h. The excess fluid was removed, and the residue was washed several times and permitted to deposit for 30 min. The remnant was collected by vacuum filtration and dehydrated for 2 h at 110 °C. The resultant Mn_3_O**_4_**–CS composite is hereafter denoted as S1.

### 2.3. ZnMn_2_O_4_–CS Synthesis

MnCl_2_ and zinc chloride (ZnCl_2_) with equal *w/v* (0.5%, 1.0%, 1.5%, and 2.0%) and purified chitin (0.25 g) liquefied 2% AA were mixed in a beaker with constant magnetic stirring; the beaker was then transferred to a hotplate at 70 °C for 2 h. Freshly prepared NaOH solution (45% *w*/*v*) was added dropwise until the mixture formed a brown/black precipitate, which was allowed to settle at room temperature for 24 h. The excess fluid was removed, and the excess fluid was washed several times and allowed to deposit for 30 min. The remnant was collected by vacuum filtration and dehydrated for 2 h at 110 °C. The equal *w*/*v* concentrations of 0.5%, 1.0%, 1.5%, and 2.0% for MnCl_2_ and ZnCl_2_ were used to prepare the ZnMn_2_O_4_–CS composites as given in Table 1, labeled as S2, S3, S4, and S5, respectively. The final ZnMn_2_O_4_–CS composite products were stored for further characterization.

### 2.4. Characterization

The synthesized nanocomposites properties were characterized by X-ray diffraction (XRD, X’Pert PRO, PANalytical, The Netherlands) with Cu Kα radiation (*λ* = 0.15406 nm); Fourier transform infrared (FTIR) spectroscopy (Thermo-Nicolet-380, Thermo Fisher, Madison, WI, USA); photoluminescence (PL) spectroscopy, (Varian Cary Eclipse, CA, USA); UV–vis spectroscopy (2401 PC, Shimadzu, Kyoto, Japan); and scanning electron microscopy (SEM, Hitachi-S3000 H, Hitachi, Tokyo, Japan).

### 2.5. Antibacterial Activity

The antibacterial properties of the hybrid composites were investigated using the diffusion technique in sterilized Mueller–Hinton agar (MHA) medium. Different organisms (i.e., *B. subtilis*, *S. Typhi*, *E. coli*, and *P. aeruginosa*) were rubbed onto the surface of MHA broth and cultured. Nanocomposites S1–S5 were added to various regions of the plates. Thereafter, the MHA broth plates were nurtured for 24 h at 37 °C and the zone of inhibition was estimated in millimeters. The micro dilution process experiments were carried out to assess the minimum inhibitory concentrations (MICs) for the hybrid composites. 96-well disposable plates were used for the micro dilution process. The serial dilutions of nanocomposites antibacterial agents in the liquid media were added to the microdilution trays bacterial suspension (~10^5^ colony-forming unit/mL) and the plates were nurtured at 37 °C for 48 h. MICs were assessed as the lowest concentration of nanocomposites to inhibit the growth of the organisms. The antibacterial experiments were carried out in triplicate; their mean results are described with standard deviation (SD). The statistical one-way analysis of variance (ANOVA) was calibrated by Student’s *t*-test with significance of *p* ≤ 0.05. The statistical values were appraised by SPSS software, IBM, NY, USA (version 13.0).

## 3. Results and Discussion

**Figure 1:**
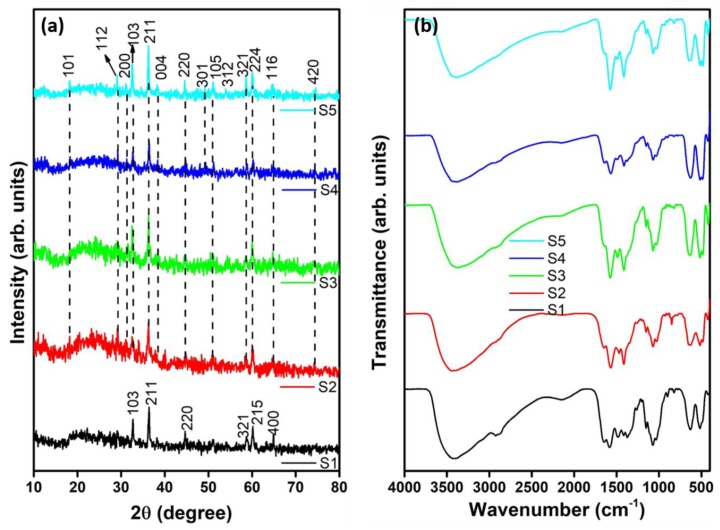
(**a**) XRD and (**b**) FTIR profiles of nanocomposites S1–S5.

A simple sol–gel chemical reaction was used to synthesize the Mn_3_O_4_–CS and ZnMn_2_O_4_–CS nanocomposites as schematically shown in Figure S1, supporting information. The formation of nanocomposites and their structural characteristics were experimentally investigated by XRD analysis. Figure 1a shows the XRD profiles of nanocomposites S1–S5. The XRD pattern of the S1 (Mn_3_O_4_–CS) nanocomposite shows reflections indexed to the (103), (211), (220), (321), (215), and (400) planes of tetragonal-structured hausmannite, Mn_3_O_4_ (JCPDS: 80-0382). The observed pattern confirms the (211) preferred orientation of the nanosized crystallites. The XRD patterns of the ZnMn_2_O_4_–CS nanocomposites (S2–S5) show reflections indexed to the (101), (112), (200), (103), (211), (004), (220), (301), (105), (312), (321), (224), (116), and (420) lattice orientations. In addition, the peak associated with the (211) lattice orientation is particularly intense in the patterns of all of the ZnMn_2_O_4_–CS nanocomposites, indicating their preferred orientation. The observed XRD peaks are well indexed to the body-centered tetragonal structure of ZnMn_2_O_4_ (JCPDS: 77-0470). The XRD reflection intensities changed slightly as the *w/v* ratio of the metal sources was varied; however, the structure of the ZnMn_2_O_4_ compound was not altered. The XRD results clearly indicate the arrangements of metal atoms in the nanocomposites.

FTIR analysis was used to characterize the Mn_3_O_4_–CS and ZnMn_2_O_4_–CS nanocomposites. Figure 1b shows the FTIR profiles of nanocomposites S1–S5. The FTIR spectrum of the S1 nanocomposite shows a wide O–H stretching vibration at 3402 cm^−1^ [4]. The alkyl stretching and C–O–C vibrations are located at 2873 and 2922 cm^−1^, respectively, the spectrum of the **S1** composite [41,42]. The vibration at ~1647 cm^−1^ is attributed to the characteristic behavior of Mn (from Mn_3_O_4_) interacting with C=O groups [43]. A characteristic C–C stretching vibration is observed at 1583 cm^−1^ [44]. For the **S1** nanocomposites, the peaks at 1483 and 1415 cm^−1^ are attributed to the symmetric stretching of CH_3_ groups and C–H bending vibrations, respectively [29,44]. Peaks at 1373, 1259, and 1151 cm^−1^ are attributed to the –CH_3_ out-of-plane, OH in-plane, and C–N in-plane bending vibrations, respectively [43]. The C–H in-plane bending vibration peaks are observed at 1073 and 1032 cm^−1^ [45]. The peaks associated with C–O ring stretching and polysaccharide structure groups are observed at 893 and 820 cm^−1^, respectively [46,47]. In the spectrum of S1, Mn–O vibrations are observed in the region 630–427 cm^−1^ [48]. The tetragonal lattice vibrations of Mn_3_O_4_, consistent with the XRD analysis, in S1 is confirmed by the peak at 525 cm^−1^ [49]. For the ZnMn_2_O_4_–CS nanocomposites (S2–S5), the C–C stretching and C–H bending vibrations are shifted to ~1575 and 1411 cm^−1^, respectively, and their intensities are increased. The Mn_3_O_4_ tetragonal lattice vibrations are shifted to 521 cm^−1^, whereas the ZnO vibrations are located at ~480 and 423 cm^−1^ in the spectra of the S2–S5 nanocomposites [4,50]. The FTIR and XRD results confirmed the successful preparation of ZnMn_2_O_4_–CS nanocomposites.

**Figure 2:**
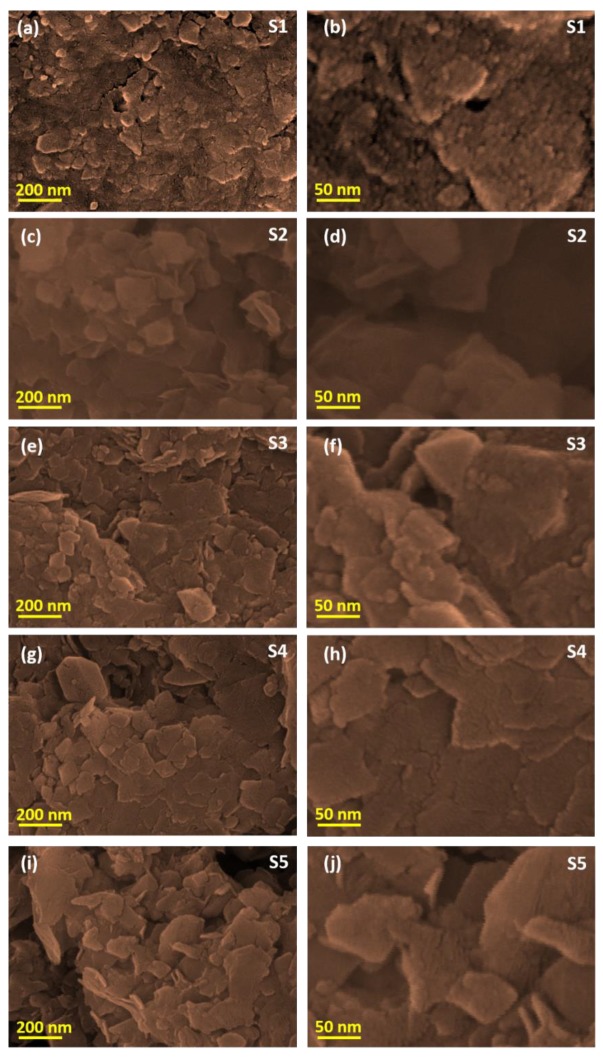
SEM micrographs of composites (**a**,**b**) S1, (**c**,**d**) S2, (**e**,**f**) S3, (**g**,**h**) S4, and (**i**,**j**) S5.

We used SEM to intensively investigate the effects of various metal oxide concentrations on the surface morphological properties of the hybrid nanocomposites. To illustrate the morphology variation in the nanocomposites, see the pure CS SEM micrograph provided in the Figure S2. Figure 2 shows SEM micrographs of the Mn_3_O_4_–CS (S1) and ZnMn_2_O_4_–CS (S2–S5) nanocomposites. Spherically structured patch-shaped agglomerated grains are observed in the micrographs of the S1 nanocomposite (Figure 2a). Hillocks and voids are clearly observed in the SEM micrographs. The higher-magnification images show that smaller-sized spherical grains produced the larger-sized grains (Figure 2b). For ZnMn_2_O_4_–CS with equal 0.5% *w/v* concentrations of Zn and Mn sources in the S2 composite, grain clusters are clearly observed with uneven morphology in the lower-magnification micrograph (Figure 2c). Rose-petal-structured grains were observed in the higher-magnification micrograph of the S2 nanocomposite, as shown in the Figure 2d. For the S3 composite prepared with the 1.0% (*w*/*v*) Zn and Mn sources, sheet-like agglomerated grains with highly textured morphology are observed (Figure 2e). High-resolution images support the existence of sheet-like, tightly bound grain bunches (Figure 2f). When the source concentration was increased to 1.5% (*w*/*v*) to synthesize the S4 hybrid composite (Figure 2g), sheet-like grains similar to those in the S3 nanocomposites were observed; their interconnected nanosheet grains were captured in the higher-magnification images (Figure 2h). Rod- and sheet-like morphologies are observed in the S5 nanocomposite, as shown in Figure 2i,j. Moreover, the high-resolution images (Figure 2j) show nanorods protruding from the sheet-like grains. These SEM observations reveal enormous morphological tuning through variation of the metal oxide matrix ratio in the ZnMn_2_O_4_–CS nanocomposites.

**Figure 3:**
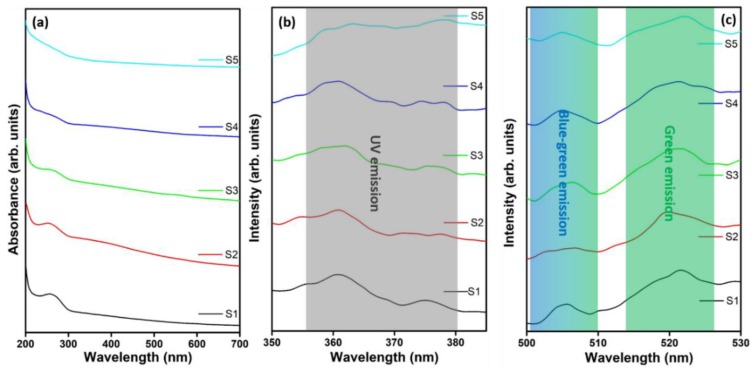
(**a**) UV–vis and (**b**,**c**) PL spectra of nanocomposites S1–S5.

The hybrid composites were further analyzed by UV–vis spectroscopy. Figure 3a displays the UV–vis spectra for nanocomposites S1–S5. The observed broad absorption peak at 265 nm for S1 is related to the *n* → σ^∗^ transport of the CS amino group [40,46]. The absorption peak intensity decreases linearly with increasing metal matrix ratio of the Mn and Zn sources used to synthesize nanocomposites S2–S5, which is strongly correlated with our earlier observations [4]. The PL spectra of the Mn_3_O_4_–CS and ZnMn_2_O_4_–CS nanocomposites were recorded using a 341 nm excitation source. Figure 3b,c shows the PL spectra of nanocomposites S1–S5. The results reveal UV (~360 nm and ~375 nm), blue (~505 nm), and green (~520 nm) emission bands [51,52]. The electron–hole recombination and free exciton emission by an exciton–exciton impact result in the near-band edge of UV emission [53]. The observation of a broad peak at approximately 360 nm with a low-intensity shoulder peak at approximately 376 nm is attributed to the interaction of CS ions with the metal oxide lattice (Figure 3b) [52]. Moreover, blue-green band emission peaks situated at ~505 nm in the spectra of S1–S5 are attributed to oxygen vacancies (Figure 3c) [54]. The wide green emission band that appears at approximately 520 nm is attributed to trap states in the conduction and valence bands, which can be established by surface defects or surface dangling bonds on the surface of hausmannite nanostructures [55]. The observed optical and luminescence results further validate the variation of surface properties through alteration of the metal matrix ratio in the nanocomposites.

**Figure 4:**
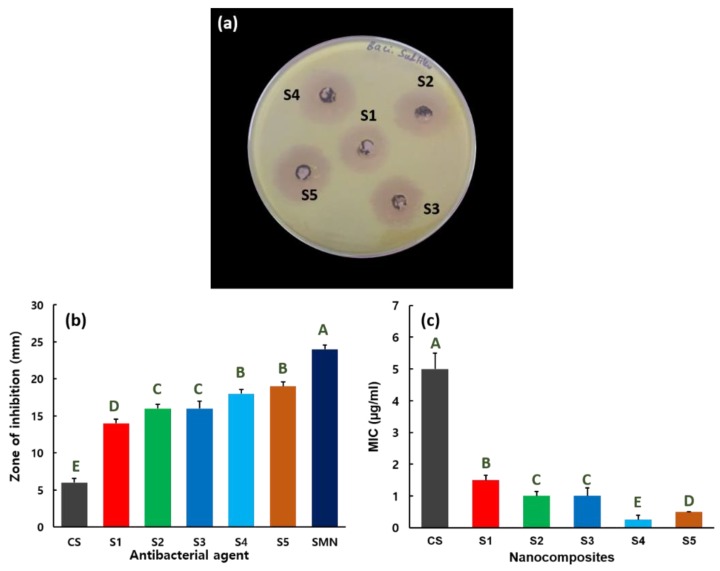
(**a**) Antibacterial activity of nanocomposites S1–S5 versus *B. subtilis*. (**b**) Zone of inhibition for chitosan (CS), streptomycin (SMN) and nanocomposites S1–S5, stated as mean ± SD from the results of triplicate tests. Statistical analysis indicated that the values are significantly (*p* ≤ 0.05) varied between their experiments, which are indicated by capital letters. (**c**) Minimum-inhibitory-concentration (MIC) values for CS and nanocomposites S1–S5 versus *B. subtilis*, expressed as mean ± SD from the triplicate tests. Statistical analysis indicated that the values are significantly (*p* ≤ 0.05) varied between their experiments, which are indicated by capital letters.

Detailed antibacterial activities of nanocomposites S1–S5 (Mn_3_O_4_–CS and ZnMn_2_O_4_–CS) were measured against *B. subtilis*, *S. typhi*, *E. coli*, and *P. aeruginosa* pathogens. For the comparison, the standard antibiotic of streptomycin (20 µg), further noted as SMN, and different CS concentrations were used as the antibacterial agent and their results are incorporated. Figure 4a shows the antibacterial properties of S1–S5 against *B. subtilis* Gram-positive pathogen by zone of inhibition assay. Agar plate represents the SMN and CS antibacterial activity against *B. subtilis* shown in the Figure S3. The zone of inhibition versus *B. subtilis* for CS, SMN, and nanocomposites S1–S5 are given in Figure 4b. The zones of inhibition are 24, 6, 13, 16, 16, 18, and 19 mm for SMN, CS, S1, S2, S3, S4, and S5, respectively, against *B. subtilis* bacteria. The antibacterial activity is effectively enhanced with increasing source concentrations used to prepare the ZnMn_2_O_4_–CS nanocomposites, as validated in Figure 4a,b. The improved antibacterial performance against *B. subtilis* was indicated by a 19 mm of zone of inhibition for the **S5** nanocomposite. Figure 4c displays the MIC values for the CS and S1–S5 nanocomposites against *B. subtilis*. A low 0.5 µg/mL MIC is estimated for the S5 nanocomposite versus *B. subtilis*. The results reveal that small amounts of the S5 nanocomposite can effectively inhibit the growth of *B. subtilis.* The CS exposed the 5 µg/mL MIC versus *B. subtilis*.

**Figure 5:**
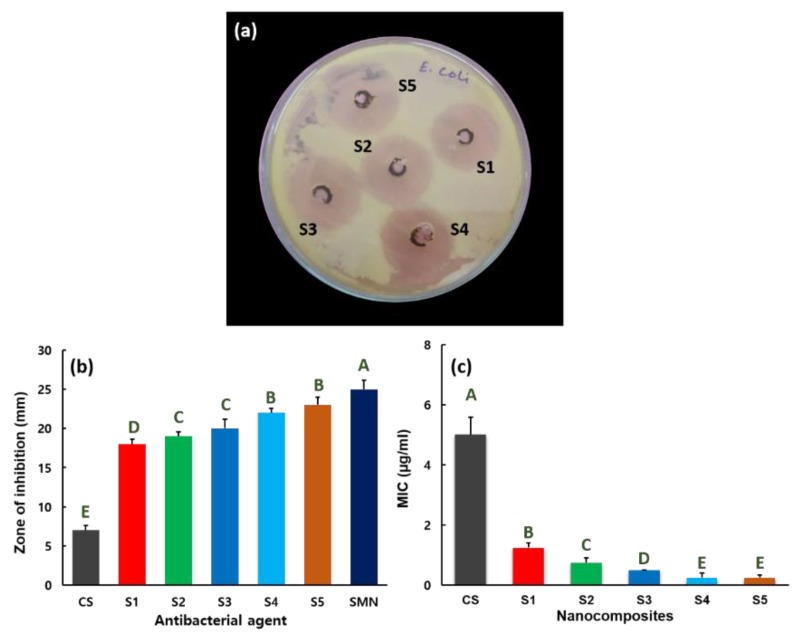
(**a**) Antibacterial activity of nanocomposites S1–S5 versus *E. coli* organism. (**b**) Zone of inhibition for CS, SMN, and nanocomposites S1–S5, stated as mean ± SD from the results of triplicate tests. Statistical analysis indicated that the values are significantly (*p* ≤ 0.05) varied between their experiments which are indicated by capital letters. (**c**) MIC values for CS and nanocomposites S1–S5 versus *E. coli*, expressed as mean ± SD from the triplicate tests. Statistical analysis indicated that the values are significantly (*p* ≤ 0.05) varied between their experiments, which are indicated by capital letters.

The antibacterial behaviors of nanocomposites S1–S5 versus *E. coli* Gram-negative bacteria are shown in Figure 5. The synthesized nanocomposites inhibit/destroy the growth of the *E. coli* pathogen proficiently, as validated in Figure 5a. For comparison, SMN and CS antibacterial activity against *E. coli* is provided in Figure S4. Nanocomposites S1–S5 produce the zones of inhibition of 18, 19, 20, 22, and 23 mm, respectively, against the *E. coli* pathogen (Figure 5b), whereas SMN and CS create the 25 and 7 mm, respectively. Figure 5c shows the MIC parameters of the CS and S1–S5 hybrid nanocomposites versus *E. coli* bacteria. Nanocomposites S1–S5 prevent the development of *E. coli*, with MICs of 1.25, 0.75, 0.50, 0.25, and 0.25 µg/mL, respectively. The maximum MIC is 5.0 µg/mL against *E. coli* for CS. A 0.25 µg/mL MIC value versus *E. coli* is estimated for hybrids S4 and S5, which demonstrates the importance of the increased metal matrix ratio in synthesizing the ZnMn_2_O_4_–CS composites.

**Figure 6:**
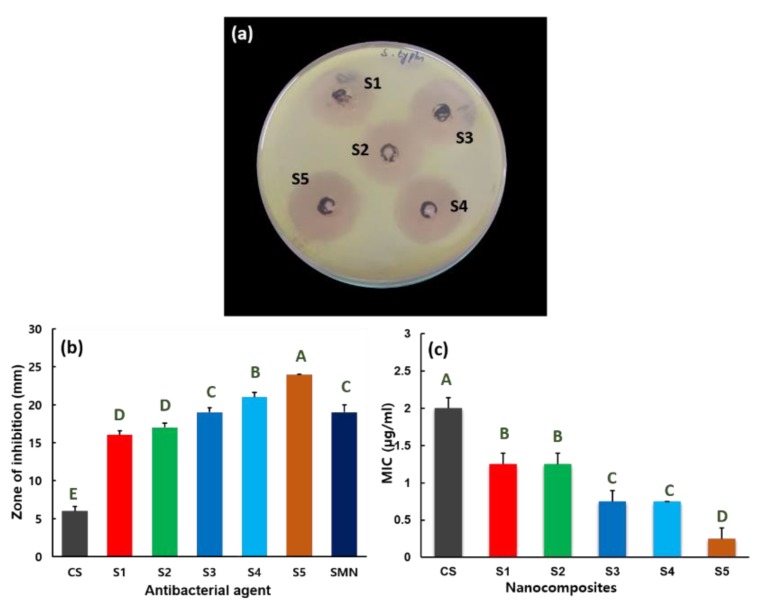
(**a**) Antibacterial activity of S1–S5 nanocomposites versus *S. typhi* organism. (**b**) Zone of inhibition for CS, SMN, and nanocomposites S1–S5, stated as mean ± SD from the results of triplicate tests. Statistical analysis indicated that the values are significantly (*p* ≤ 0.05) varied between their experiments, which are indicated by capital letters. (**c**) MIC values for CS and nanocomposites S1–S5 versus *S. typhi*, expressed as mean ± SD from the triplicate tests. Statistical analysis indicated that the values are significantly (*p* ≤ 0.05) varied between their experiments, which are indicated by capital letters.

Figure 6 displays the inhibiting rate of nanocomposites S1–S5 for *S. typhi*. The experimental results clearly demonstrate inhibition behavior against *S. typhi* propagation for nanocomposites S1–S5 (Figure 6a). The agar plate image to expose the antibacterial activity of SMN and CS against *S. typhi* is provided in the Figure S5. Inhibition zones of 19, 6, 16, 17, 19, 21, and 24 mm are observed for SMN, CS, S1, S2, S3, S4, and S5, respectively, against *S. typhi* (Figure 6b). Figure 6c illustrates the MIC range for CS and nanocomposites S1–S5 versus *S. typhi* bacteria. The nanocomposites S1–S5 prevent the growth of *S. typhi*, with MICs of 1.25, 1.5, 1.0, 0.75, and 0.25 µg/mL, respectively. The experimental results confirm that the **S5** nanocomposite exhibits efficient antibacterial activity toward *S. typhi*, with a small MIC parameter of 0.25 µg/mL. For CS, the 2.0 µg/mL MIC value is against *S. typhi*.

**Figure 7:**
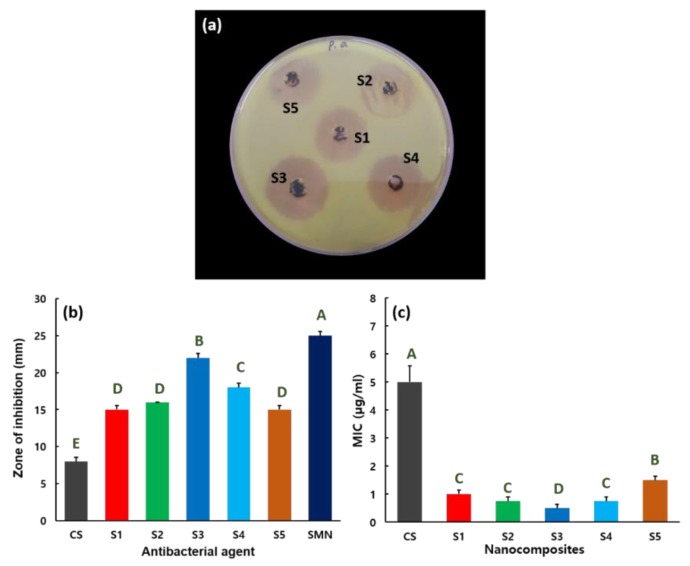
(**a**) Antibacterial activity of nanocomposites S1–S5 nanocomposites versus *P. aeruginosa*. (**b**) Zone of inhibition for CS, SMN, and nanocomposites S1–S5, with mean ± SD from triplicate tests. Statistical analysis indicated that the values are significantly (*p* ≤ 0.05) varied between their experiments, which are indicated by capital letters. (**c**) MIC values for CS and nanocomposites S1–S5 versus *P. aeruginosa*, with mean ± SD from triplicate tests. Statistical analysis indicated that the values are significantly (*p* ≤ 0.05) varied between their experiments, which are indicated by capital letters.

The antibacterial activity of nanocomposites S1–S5 against *P. aeruginosa* bacterial is shown in Figure 7. The agar plate image used to evaluate the antibacterial properties of nanocomposites S1–S5 versus *P. aeruginosa* is shown in Figure 7a. The antibacterial activity of SMN and CS against *P. aeruginosa* is given in the Figure S6. The zones of inhibition for CS, SMN, S1, S2, S3, S4, and S5 against the *P. aeruginosa* pathogen were 8, 25, 15, 16, 22, 18, and 15 mm, respectively (Figure 7b). Enhanced antibacterial activity against *P. aeruginosa* was observed for the S3 composite, with the zone of inhibition of 22 mm, possibly because of surface modification. Figure 7c shows the MIC values of nanocomposites S1–S5 against *P. aeruginosa* bacteria. Nanocomposites S1–S5 exhibit MICs of 1.0, 0.75, 0.50, 0.75, and 1.50 µg/mL, respectively, to prevent the growth of *P. aeruginosa*. A smaller MIC of 0.5 µg/mL versus *P. aeruginosa* is estimated for the S3 hybrid, consistent with its zone of inhibition performance. The effectiveness of the S3 ZnMn_2_O_4_–CS composite is attributed to its surface properties. The maximum MIC of 5.0 µg/mL is perceived for CS versus *P. aeruginosa*.

The antibacterial activity of the metal-oxide-incorporated CS nanocomposites can be explained by the following mechanism [4,56,57]. The bactericidal properties of CS are related to the positive charge of CS and negative charge of the cell wall of the pathogen, which result in cell wall lysis and cytoplasmic discharge, leading to the mortality of the microorganism [57,58]. The antibacterial bactericidal process is greatly improved for metal oxide–CS composites because of the enhanced chelation behavior of the hybrid surface with the bacterial cell, which leads to an increase in the death rate of bacteria. In addition, the structure and charge dissemination of the hybrids are correlated with the pathogen cell wall, which eases the dispersion of the antibacterial agent into the bacterial cell and enhances the lipophilic behavior of chelation in addition to the interaction between the metal ions and the membrane. The bonded complexes readily deactivate the enzyme, changing the metabolic mechanism of bacteria [59]. We presumed that the aforementioned geometrical modifications disturb the cell diffusion and prevent typical cell progression.

## 4. Conclusions

A one-pot simple sol–gel chemical reaction was used to prepare Mn_3_O_4_–CS and ZnMn_2_O_4_–CS nanocomposites. XRD and FTIR analyses confirmed the formation of Mn_3_O_4_–CS and ZnMn_2_O_4_–CS nanocomposites. Morphological alterations were observed for nanocomposites S1–S5 by SEM analyses at different magnifications. The modified morphological and structural properties effectively altered the optical and luminescence results for nanocomposites S1–S5. The antibacterial activity of Mn_3_O_4_–CS and ZnMn_2_O_4_–CS were tested against Gram-positive organism *B. subtilis* and Gram-negative organism *E. coli*, *P. aeruginosa,* and *S. typhi.* Moreover, the ZnMn_2_O_4_–CS nanocomposite demonstrated superior immunoactivity against all of the microorganisms. The S5 ZnMn_2_O_4_-CS nanocomposite exhibited enhanced activities with low MIC values against *B. subtilis, E. coli*, and *S. typhi* bacteria, whereas the S3 nanocomposite exhibited enhanced activity against *P. aeruginosa*. Hence, the results illustrate the significance of ZnMn_2_O_4_–CS nanocomposites and their highly inhibitory properties against various pathogens.

## Figures and Tables

**Table 1:** The chemical source ratios for the Mn_3_O_4_–CS and ZnMn_2_O_4_–CS composites.

Nanocomposites	Source			Label
	Chitin (g)	MnCl_2_ (*w*/*v*) %	ZnCl_2_ (*w*/*v*) %	
Mn_3_O_4_–CS	0.25	0.5	0	S1
ZnMn_2_O_4_–CS	0.25	0.5	0.5	S2
ZnMn_2_O_4_–CS	0.25	1.0	1.0	S3
ZnMn_2_O_4_–CS	0.25	1.5	1.5	S4
ZnMn_2_O_4_–CS	0.25	2.0	2.0	S5

## Supplementary Materials

The following are available online at https://www.mdpi.com/2079-4991/9/11/1589/s1, Figure S1: (a) Schematic showing the synthesis of ZnMn_2_O_4_–CS nanocomposites by sol-gel chemical reaction and their nanopowder; (b) typical mechanism of antibacterial activity of the nanocomposites, Figure S2: SEM micrograph of CS, Figure S3: Antibacterial activity of Streptomycin and CS versus *B. subtilis*, Figure S4: Antibacterial activity of Streptomycin and CS versus *E. coli*, Figure S5: Antibacterial activity of Streptomycin and CS versus *S. typhi*, Figure S6: Antibacterial activity of Streptomycin and CS versus *P. aeruginosa*.
