# Supplementary material for: Synthesis and Antibacterial Properties of Novel ZnMn2O4–Chitosan Nanocomposites
Source: Nanomaterials (Basel). 2019 Nov 9;9(11):1589. doi: 10.3390/nano9111589 (PMC6915490; doi:10.3390/nano9111589)
Supplement: Supplementary file 1 [file nanomaterials-09-01589-s001.pdf]

Supporting information

Synthesis and antibacterial properties of novel $ZnMn_2O_4$–chitosan nanocomposites

Rajiv Gandhi Packirisamy[1], Chandramohan Govindasamy[1], Anandhavelu Sanmugam[2], K. Karuppasamy[3], Hyun-Seok Kim[3], Dhanasekaran Vikraman[3*]

[1]Research and Development Centre, Bharathiar University, Coimbatore 641046, India.
[2]Department of Chemistry, Vel Tech Multi Tech., Chennai 600062, India.
[3]Division of Electronics and Electrical Engineering, Dongguk University-Seoul, Seoul 04620, Republic of Korea.
*Correspondence: v.j.dhanasekaran@gmail.com; Tel.: +82-2-2260-8735

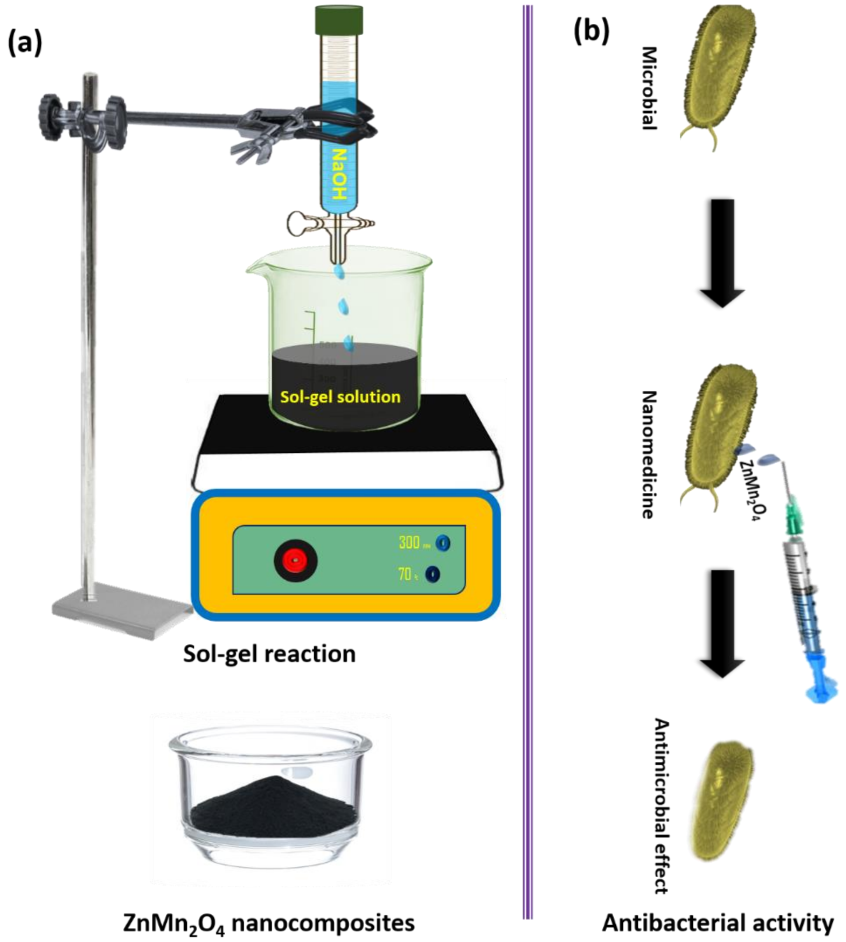

Figure S1. (a) Schematic showing the synthesis of $ZnMn_2O_4$–CS nanocomposites by sol–gel chemical reaction and their nanopowder; **(b)** typical mechanism of antibacterial activity of the nanocomposites.

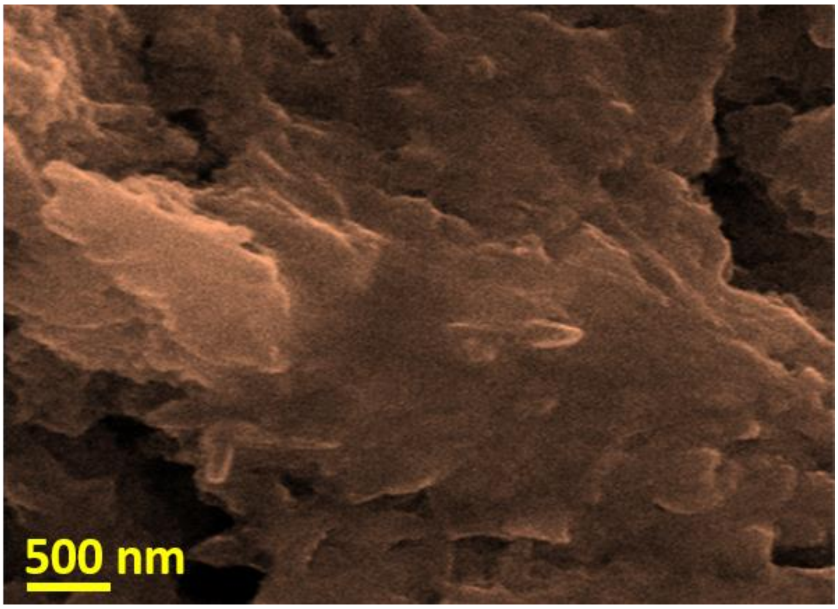

Figure S2. SEM micrograph of CS

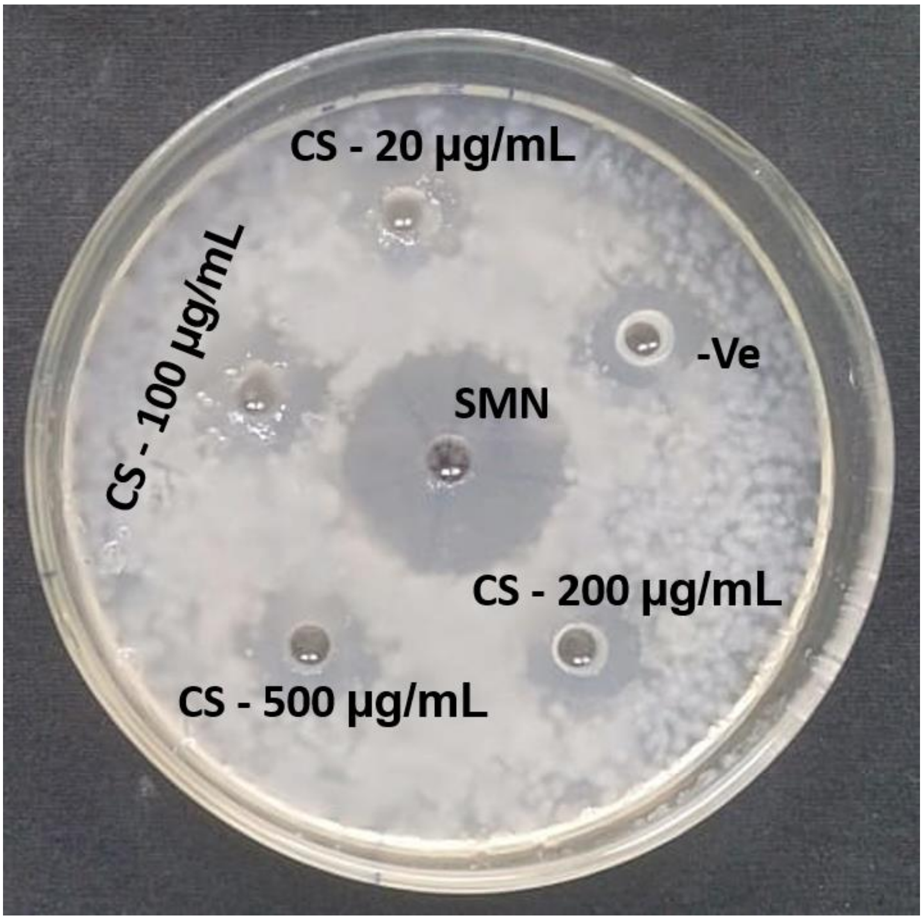

Figure S3. Antibacterial activity of Streptomycin and CS versus *B. subtilis*.

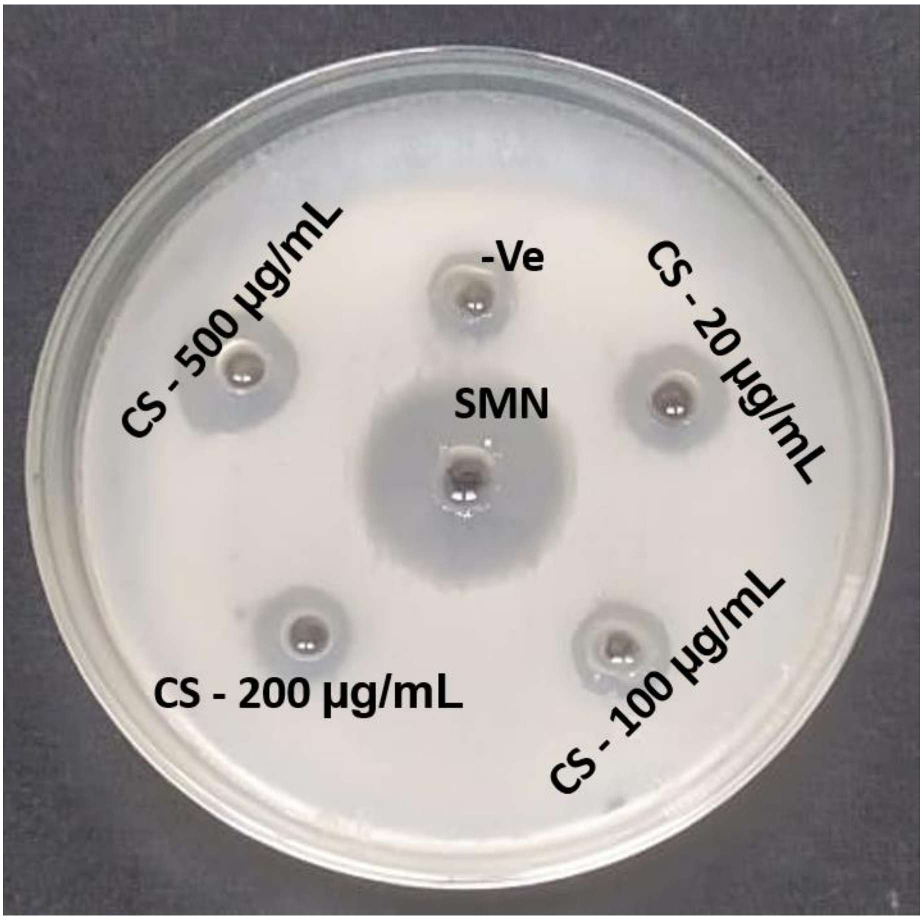

Figure S4. Antibacterial activity of Streptomycin and CS versus *E. coli*.

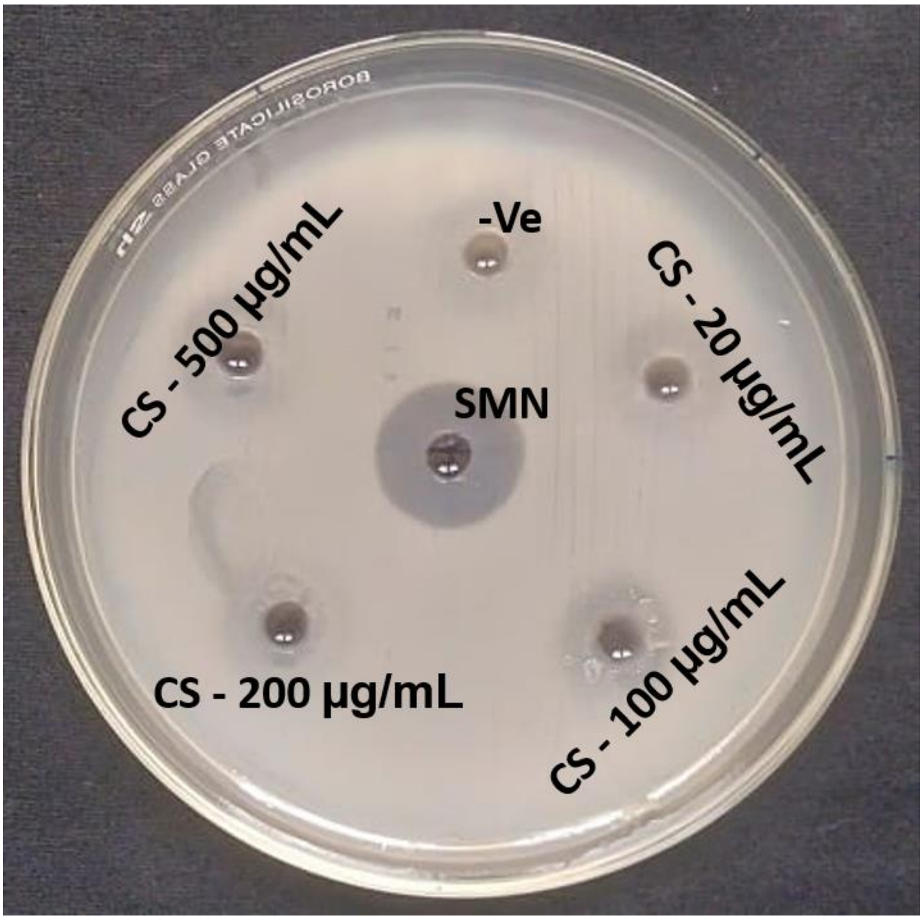

Figure S5. Antibacterial activity of Streptomycin and CS versus *S. typhi*.

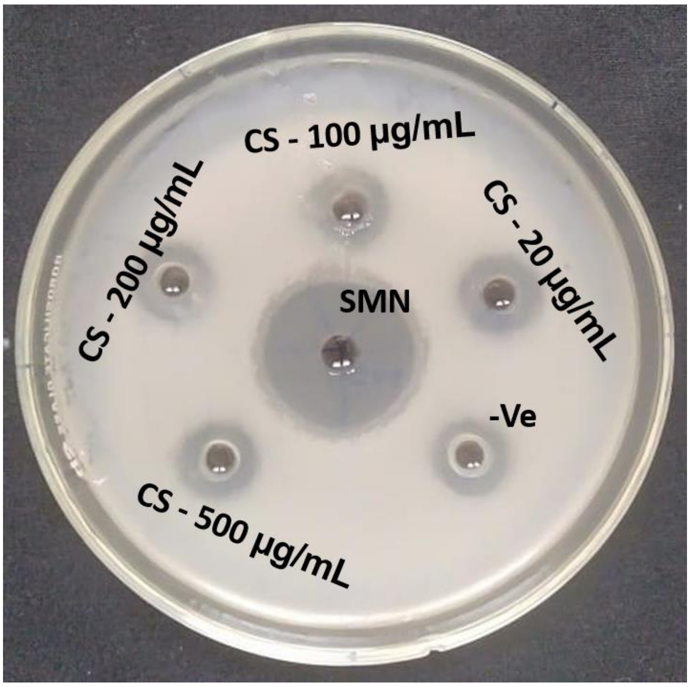

Figure S6. Antibacterial activity of Streptomycin and CS versus *P. aeruginosa*.
